# Mitochondrial tRNA processing defects reprogram mitochondrial and cellular homeostasis

**DOI:** 10.1016/j.jbc.2025.110334

**Published:** 2025-06-03

**Authors:** Gao Zhu, Yunfan He, Xincheng Li, Yun Xiao, Huisen Zhan, Maoli Duan, Min-Xin Guan

**Affiliations:** 1 Center for Mitochondrial Biomedicine and Department of Otolaryngology-Head and Neck Surgery, The Fourth Affiliated Hospital, Zhejiang University School of Medicine, Yiwu, Zhejiang, China; 2 Institute of Genetics, Zhejiang University International School of Medicine, Hangzhou, Zhejiang, China; 3 Center for Genetic Medicine, Zhejiang University International Institute of Medicine, Yiwu, Zhejiang, China; 4 Department of Otolaryngology Head and Neck Surgery & Audiology and Neurotology, Karolinska University Hospital, Stockholm, Sweden; 5 Division of Mitochondrial Biomedicine, Zhejiang Provincial Key Laboratory of Genetic and Developmental Disorders, Hangzhou, Zhejiang, China

**Keywords:** mitochondrial RNA, mutation, deafness, oxidative phosphorylation, mitochondrial dynamics, mitophagy, mitochondrial unfolded protein response, autophagy process, aberrant RNA processing, mitochondrial and cellula

## Abstract

Mitochondrial tRNA processing defects have been associated with some clinical presentations including deafness. Especially, a deafness-linked m.7516delA mutation impaired the 5′ end processing of RNA precursors and mitochondrial translation. In this study, we investigated the mechanism by m.7516delA mutation-induced deficiencies mitigate organellar and cellular integrity. The m.7516delA mutation downregulated the expression of nucleus-encoding subunits and upregulated assembly factors of complex IV and altered the assembly and activities of oxidative phosphorylation (OXPHOS) complexes. The impairment of OXPHOS alleviated mitochondrial quality control processes, including the imbalanced mitochondrial dynamics *via* increasing fission with abnormal mitochondrial morphology. The m.7516delA mutation upregulated both ubiquitin-dependent and independent mitophagy pathways, evidenced by increasing levels of Parkin, BNIP3, NIX, and MFN2-ubiquitination and altering interaction between MFN2 and MUL1 or Parkin, to facilitate the degradation of severely damaged mitochondria. Strikingly, the m.7516delA mutation activated the integrated stress response (ISR) pathway, evidenced by upregulation of GCN2, P-GCN2, p-eIF2**α**, CHOP, ATF4, and elevated the nuclear location of ATF5 to minimize the damages in defective mitochondria. Both activation of the ISR and PINK1/Parkin mitophagy pathways ameliorate the cell homeostasis *via* elevating the autophagy process and upregulating apoptotic pathways. Our findings provide new insights into underlying aberrant RNA processing-induced dysfunctions, reprogrammed organelles, and cellular integrity.

The human cochlea is a high-energy-demanding tissue that plays a vital role in the hearing function. Healthy human mitochondria, to produce cellular energy through oxidative phosphorylation (OXPHOS), are important for cochlear cell function and normal hearing.

Sensorineural deafness often occurs as a consequence of damaged or deficient cochlear hair cells or spiral ganglion cells in the auditory nerve, both of which serve the sense of hearing by transmitting the sound waves from the cochlea to the brain (1, 2, 3, 4). Mutations in human mitochondrial RNA (mt-RNA) have been linked to both syndromic deafness (deafness with other medical problems such as diabetes) and nonsyndromic deafness (deafness with only clinical presentation) (1, 2, 3, 4, 5, 6, 7, 8, 9, 10). These mt-RNAs, encoded by mitochondrial genomes, are composed of 13 mRNAs encoding 13 subunits of OXPHOS complexes, and 2 rRNAs and 22 tRNAs essential for translation (11, 12). The 12S rRNA 1555A>G and 1494C>T mutations have been associated with both aminoglycoside-induced and nonsyndromic deafness in many families worldwide (13, 14, 15, 16). The syndromic deafness-associated tRNA mutations include the MELAS-associated tRNA^Leu(UUR)^ 3243A>G and MERRF-associated tRNA^Lys^ 8344A>G and maternally inherited diabetes and deafness (MIDD)-associated tRNA^Glu^ 14692A>G mutations (17, 18, 19). The nonsyndromic deafness-associated tRNA mutations are the tRNA^Ser(UCN)^ 7445A>G, and 7511T>C, tRNA^His^ 12201T>C, tRNA^Asp^ 7551A>G, tRNA^Ile^ 4295A>G, tRNA^Cyr^ 5783C>T, and m.7516delA mutations (20, 21, 22, 23, 24, 25, 26). These tRNA mutations impact their structure and function, including the processing of the tRNA from the primary transcripts, posttranscriptional modifications, stability of folded secondary structure, charging of the tRNA, or codon–anticodon interaction in the process of translation (20, 21, 22, 23, 24, 25, 26). The aberrant RNA metabolisms impaired mitochondrial translation, diminished ATP production, and overproduced reactive oxygen species (ROS) required for the cochlear functions (20, 21, 22, 23, 24, 25, 26, 27, 28, 29). These mitochondrial RNA mutations-induced dysfunctions may dysregulate the expression of nuclear genes involved in organellar and cellular homeostasis through the integrated stress response (ISR), including mitochondrial unfolded protein response (UPR^mt^) and endoplasmic reticulum unfolded protein response (UPR^ER^) as well as PINK1/Parkin mitophagy pathways (28, 29, 30, 31, 32, 33, 34, 35, 36). In fact, the ISR can restore the balance by reprogramming gene expression in response to genetic and environmental stressors, including proteostasis, viral infection, and oxidative stress (32, 33). In particular, the UPR^mt^ is a repair-driven process that induces the nucleus-encoding factors, including transcriptional factors and proteases, to restore mitochondrial function and maintain cellular homeostasis from a broad range of mitochondrial stressors (34). The activation of the PINK1/Parkin mitophagy pathway facilitates the degradation of severely damaged mitochondria through the *de novo* formation of autophagosomes that enclose the damaged mitochondria prior to their transport to lysosomes for degradation, thus maintaining cellular homeostasis (35, 36). However, the mechanism underlying mt-RNA mutations that ameliorate the organellar and cellular integrity is far less understood.

In this study, we investigated the mechanism by which mitochondrial tRNA processing defects arising from the deafness-associated m.7516delA mutation mitigate organellar and cellular integrity. The previous investigation revealed that the m.7516delA mutation at the junction between mt-tRNA^Ser(UCN)^ and mt-tRNA^Asp^ altered the 5′ end processing in the precursors of mt-tRNA^Asp^ and downstream COX2 mRNA from H-strand transcripts, as well as mt-tRNA^Ser(UCN)^ and five downstream mt-tRNAs, including mt-tRNA^Tyr^ from L-strand transcripts, and impeded mitochondrial translation and membrane potentials (21). In this study, we examined whether the m.7516delA mutation-induced deficiencies regulate the expression of nucleus-encoding components of OXPHOS complexes and impact the assembly and activity of OXPHOS complexes. Second, we assessed whether these mitochondrial dysfunctions alleviate mitochondrial quality control processes *via* mitochondrial dynamics, which are maintained through ongoing fusion and fission processes (37, 38) and upregulate the PINK1/Parkin mitophagy pathway to facilitate the selective degradation of damaged mitochondria (35, 36). Furthermore, we then examined whether the mitochondrial dysfunctions arising from the m.7516delA mutation activate cellular defense pathways including UPR^mt^ (32, 33, 34) and endoplasmic reticulum unfolded protein response (UPR^ER^) (39) and then reprogram cellular homeostasis through autophagy and apoptosis.

## Result

### Disregulation of nucleus-encoding components of OXPHOS complexes

**Figure 1:**
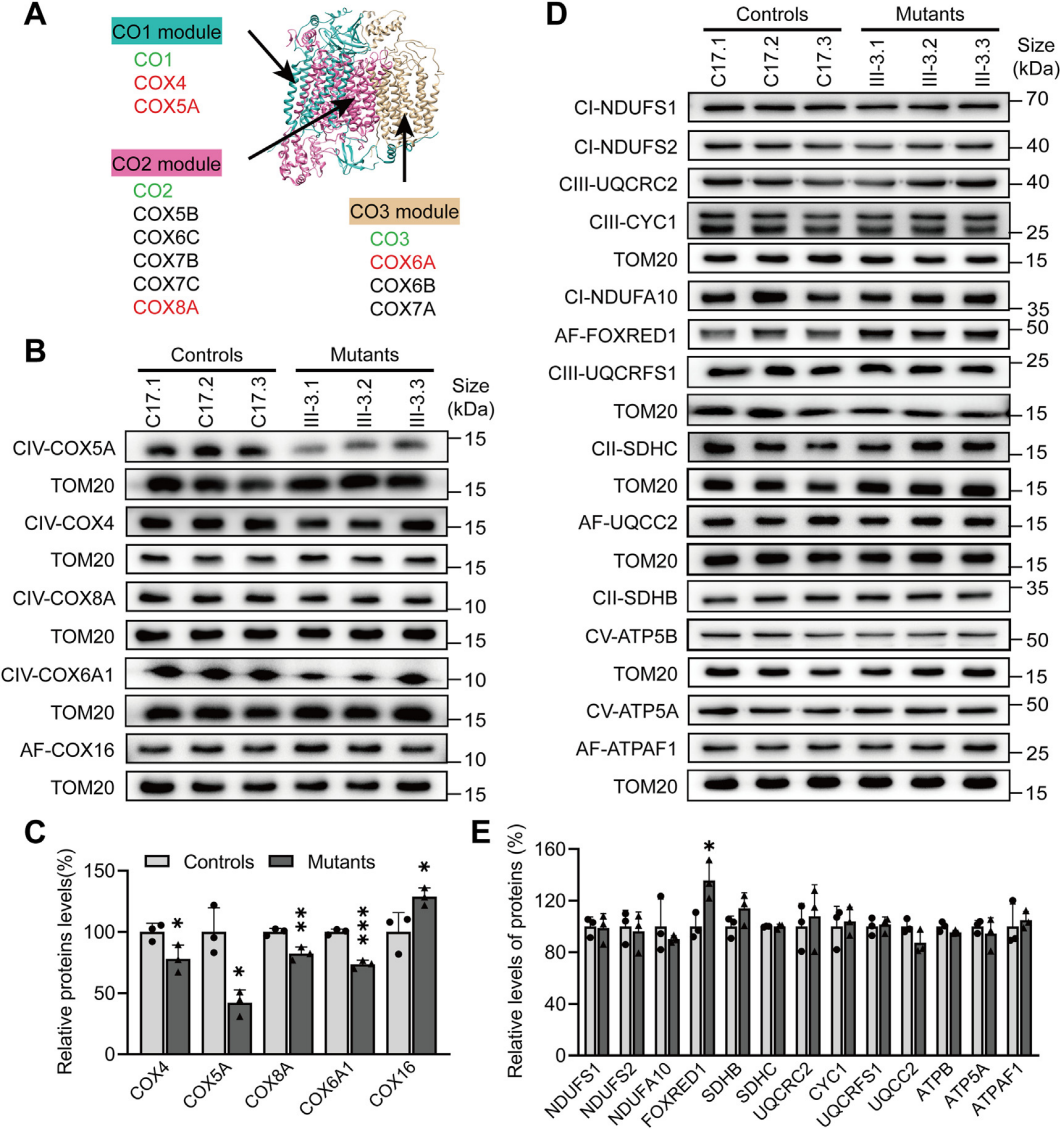
**Western blot analysis of mitochondrial proteins**. *A*, structure of complex IV (Protein Data Bank, ID 5IY5) consists of 3 mtDNA encoding subunits (*green*) and 10 nucleus-encoding subunits, 4 of which were used for this investigation (*red*). *B and D*, 20 micrograms of total cellular proteins from various cell lines were electrophoresed through a denaturing polyacrylamide gel, electroblotted, and hybridized with antibodies for nucleus-encoding subunits and assemble factors: (*B*) complex IV (COX4, COX5A, COX6A1, COX8A and COX16), (*D*) complex I (NDUFS1, NDUFS2, NDUFA10 and FOXRED1), complex II (SDHB, SDHC), complex III (CYC1, UQCRFS1, UQCRC2), and complex V (ATPB, ATP5A, ATPAF1), and TOM20 as a loading control, respectively. *C* and *E*, quantification of proteins: (*C*) Subunits and assembly factors of complex IV, (*E*) Subunits and assembly factors of complex I, II, III and V. Average relative each polypeptide content per cell was normalized to the average content per cell of TOM20 in each cell line. The values for the mutant cell lines are expressed as percentages of the values for the control cell lines. The calculations were based on three independent determinations. The error bars indicate two standard deviations (SD) of the means. *P* indicates the significance, according to the *t* test, of the differences between mutant and control cell lines. ∗*p* < 0.05; ∗∗*p* < 0.01; ∗∗∗*p* < 0.001; ∗∗∗∗*p* < 0.0001; ns, not significant.

The OXPHOS complexes consist of 13 mtDNA encoded subunits, and 72 nucleus-encoded subunits, which are synthesized in cytosol and imported into mitochondria (40). To examine whether impaired synthesis of mtDNA-encoding polypeptides due to the m.7516delA mutation regulated the expression of nucleus-encoded subunits of OXPHOS complexes, we carried out a Western blot analysis to assess the levels of 18 nuclear-encoded OXPHOS subunits in three mutant cybrids (III-3.1, III-3.2, and III-3.3) bearing the homoplasmic m.7516delA mutation derived from a Han Chinese family with maternal inheritance of deafness and three control cybrids (C17.1, C17.2, and C17.3) derived from normal hearing Chinese control subject lacking the mutation but belonging to the same mtDNA haplogroup using TOM20 as a loading control (21). These subunits included NDUFS1, NDUFS2, and NDUFA10 (subunits of complex I), SDHB and SDHC [subunits of succinate dehydrogenase (complex II)], UQCRFS1, UQCRC2 and CYC1 [subunits of ubiquinol-cytochrome *c* reductase (complex III)], COX4, COX5A, COX6A1 and COX8A (subunits of complex IV), and ATP5A and ATP5B [subunits of H^+^-ATPase (complex V)] (40). Of these, complex IV consists of 13 subunits, including CO1, CO2, and CO3, and nucleus-encoded 10 subunits (Fig. 1*A*). As shown in Figure 1, *B*–*E*, mutant cybrids exhibited marked decreases in the levels of 4 subunits of complex IV but no significant changes in the other 10 subunits of OXPHOS complexes, as compared with those in control cybrids. Especially, the levels of COX5A, COX4, COX8A, and COX6A1 in the mutant cybrids were 42%, 78%, 82%, and 74% relative to the mean values measured in the control cybrids, respectively (Fig. 1*C*). The effects of m.7516delA mutation on the biogenesis of complex IV were further supported by decreasing mRNA levels of these subunits in the mutant cybrids using qPCR technology (Fig. S1). We then examined the levels of assembly factors (FOXRED1 for complex I, UQCC2 for complex III, COX16 for complex IV, and ATPAF1 for complex V) for OXPHOS complexes by Western blot analysis (40). A shown in Figure 1, *B*–*E*, the levels of FOXRED1, UQCC2, COX16, and ATPAF1 in the mutant cybrids were 136%, 87%, 129%, and 105% relative to the mean values measured in the control cybrids, respectively. These data revealed that the m.7516delA mutation led to the downregulation of nuclear genes encoding subunits of complex IV but upregulation of these genes coding for the assembly factors of complexes I and IV.

### Defective assembly and activity of complexes I and IV

**Figure 2:**
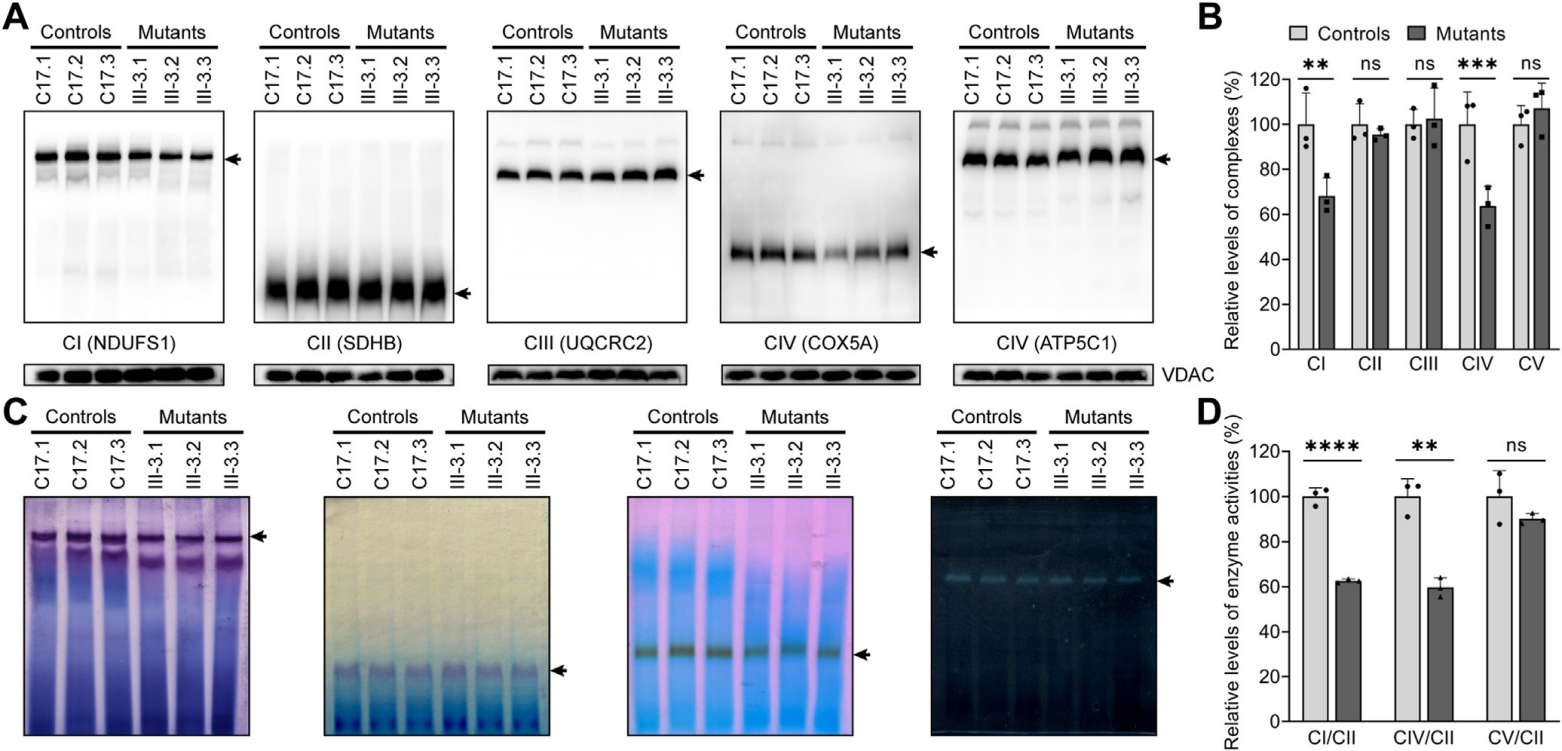
**Analysis of OXPHOS complexes**. *A*, the steady-state levels of five OXPHOS complexes. 20 micrograms of mitochondrial proteins from various cell lines were electrophoresed through BN-PAGE, electroblotted and hybridized with antibodies specific for subunits of five OXPHOS complexes (NDUFS1 for complex I, SDHB for complex II, UQCRC2 for complex III, COX5A for complex IV and ATP5C1 for complex V), and with VDAC as a loading control. *B*, quantification of levels of complexes I, II, III, IV and V in mutant and control cell lines. The calculations were based on three independent experiments. *C*, in-gel activity of complexes I, II, IV and V. The activities of OXPHOS complexes from various cell lines after BN-PAGE were measured in the presence of specific substrates [NADH and NTB for complex I, sodium succinate, phenazine methosulfate, and NTB for complex II, DAB and cytochrome *c* for complex IV, glycine, MgSO_4_, ATP and Pb (NO_3_)_2_ for complex V] (42). *D*, quantification of in-gel activities of complexes I, II, IV, and V. The calculations were based on three independent determinations in each cell line. Graph details and symbols are explained in the legend to Figure 1.

We investigated the effects of m.7516delA mutation on the stability and activities of oxidative phosphorylation machinery. Mitochondrial membrane proteins extracted from both mutant and control cybrids were separated by BN-PAGE, followed by electroblotting and hybridization with antibodies against NDUFS1, SDHB, UQCRC2, COX5A, and ATP5C (41, 42). As shown in Figure 2, *A* and *B*, the levels of complexes I, II, III, IV, and V in the mutant cybrids were 68%, 95%, 102%, 64%, and 107% of those average values in control cybrids, respectively. These data indicated that the m.7516delA mutation ablated the assembly of complexes I and IV but not other complexes. We used the in-gel activity assay to evaluate the consequence of m.7516delA mutation on the activity of complexes I, II, IV, and V. Mitochondrial membrane proteins isolated from various cell lines were separated by BN-PAGE and subsequently stained with specific substrates for the OXPHOS complexes [NADH and NTB for complex I, sodium succinate, phenazine methosulfate, and NTB for complex II, DAB and cytochrome *c* for complex IV, glycine, MgSO_4_, ATP and Pb(NO_3_)_2_ for complex V (42). As illustrated in Figure 2*C*, the defective assembly of complexes I and IV was further validated in the mutant cell lines when compared to the control cell lines. Specifically, the in-gel activities of complexes I, IV, and V in the mutant cell lines were 63%, 64%, and 90% of the average values observed in the control cybrids, respectively. In contrast, the in-gel activity of complex II in the mutant cybrids was similar to that of the control cell lines (Fig. 2*D*). These results highlighted the effect of m.7516delA mutation on the stability and activity of complexes I and IV.

### Mitochondrial dynamic imbalance toward fission

**Figure 3:**
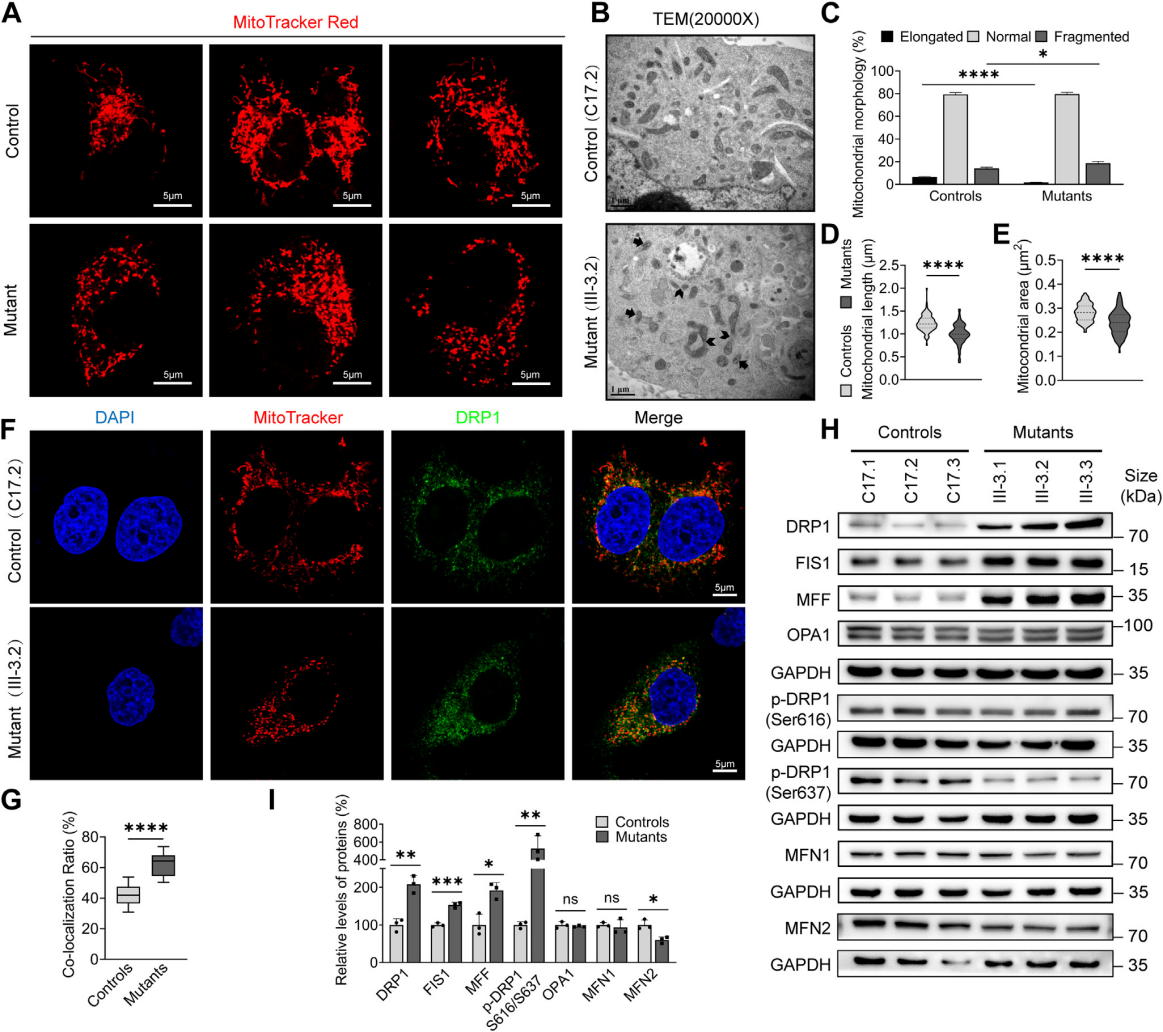
**Assessment of mitochondrial dynamics**. *A*, immunofluorescence analysis. Mitochondria from mutant cybrids (III-3.2) and control cybrids (C17.2) were visualized by immunofluorescent staining with mitochondrial dye MitoTracker analyzed by confocal microscopy. Scale bars: 10 μm. *B*, mitochondrial morphology from mutant and control cybrids examined by transmission electron microscopy. *C*, quantification of mitochondrial morphology. Mitochondrial morphology was scored as follows: Fragmented, mainly small and round; normal, mixture of round and shorter tubulated; and elongated, long and higher interconnectivity. The percentage of cells with indicated mitochondrial morphologies was determined as a percentage of the total number of cells counted (≥100 cells per experiment). n = 3 independent experiments. *D and E*, quantification of mitochondrial length (*D*), and mitochondrial area (*E*) (n ≥ 100 mitochondria counted per condition). *F*, immunofluorescent staining with mitochondrial dye MitoTracker and labeling with DRP1 antibody conjugated to Alex Fluor 488 (*green*) analyzed by confocal microscopy. Scale bars: 10 μm. *G*, quantification of colocalization of Drp1 and mitochondria. *H*, Western blot analysis of mitochondrial fission-associated proteins (DRP1, Drp1-Ser616, Drp1-Ser637, MFF, FIS1) and fusion-associated proteins (OPA1, MFN1, MFN2) among six cell lines with GAPDH as a loading control. *I*, quantification of mitochondrial fission-associated proteins and fusion-associated proteins. Three independent experiments were made for each cell line. Graph details and symbols are explained in the legend to Figure 1.

We then examined the organelle responses to the m.7516delA mutation-induced deficiencies to reshape mitochondrial dynamics, which are maintained through ongoing fusion and fission processes, as a mitochondrial quality control process (37, 38). To evaluate the effects of the m.7516delA mutation on mitochondrial morphology and dynamics, we employed immunofluorescence along with MitoTracker staining and transmission electron microscope (TEM). As illustrated in Figure 3, *A–E*, the mutant cells bearing the m.7516delA mutation exhibited abnormal mitochondrial morphology, characterized by significant increases in fragmented mitochondria and decreases in the elongated mitochondria, when compared to control cells. This suggested that the m.7516delA mutation caused an imbalance of fusion and fission. Especially, Drp1 is the major pro-fission protein whose phosphorylation at Ser616 or Ser637 promotes and inhibits the mitochondrial fission process, respectively (43, 44). As shown in Figure 3, *F–I*, both immunofluorescence and Western blot data showed markedly increasing levels of Drp1 in the mutant cells when compared to control cells. Furthermore, the levels of Drp1 with phosphorylation at Ser616 or Ser637 in mutant cybrids increased and decreased, as compared with those in control cybrids, respectively. This indicated that the m.7516delA mutation facilitated mitochondrial fission. Moreover, we assessed the levels of two fission-related proteins and three fusion-related proteins, including MFF, FIS1, OPA1, MFN1, and MFN2, in both mutant and control cell lines (37, 38). As shown in Figure 3, *H* and *I*, mutant cell lines displayed drastically increased levels of MFF and FIS1. However, the average levels of OPA1 and MFN1 in mutant cybrids were comparable with those in control cybrids, while the levels of MFN2, playing a role in mitophagy in the mutant cybrids (45), were significantly reduced when compared with those in control cybrids. These data indicated that the m.7516delA resulted in the mitochondrial dynamic imbalance toward fission.

### Upregulated ubiquitin-dependent and ubiquitin-independent mitophagy pathways

**Figure 4:**
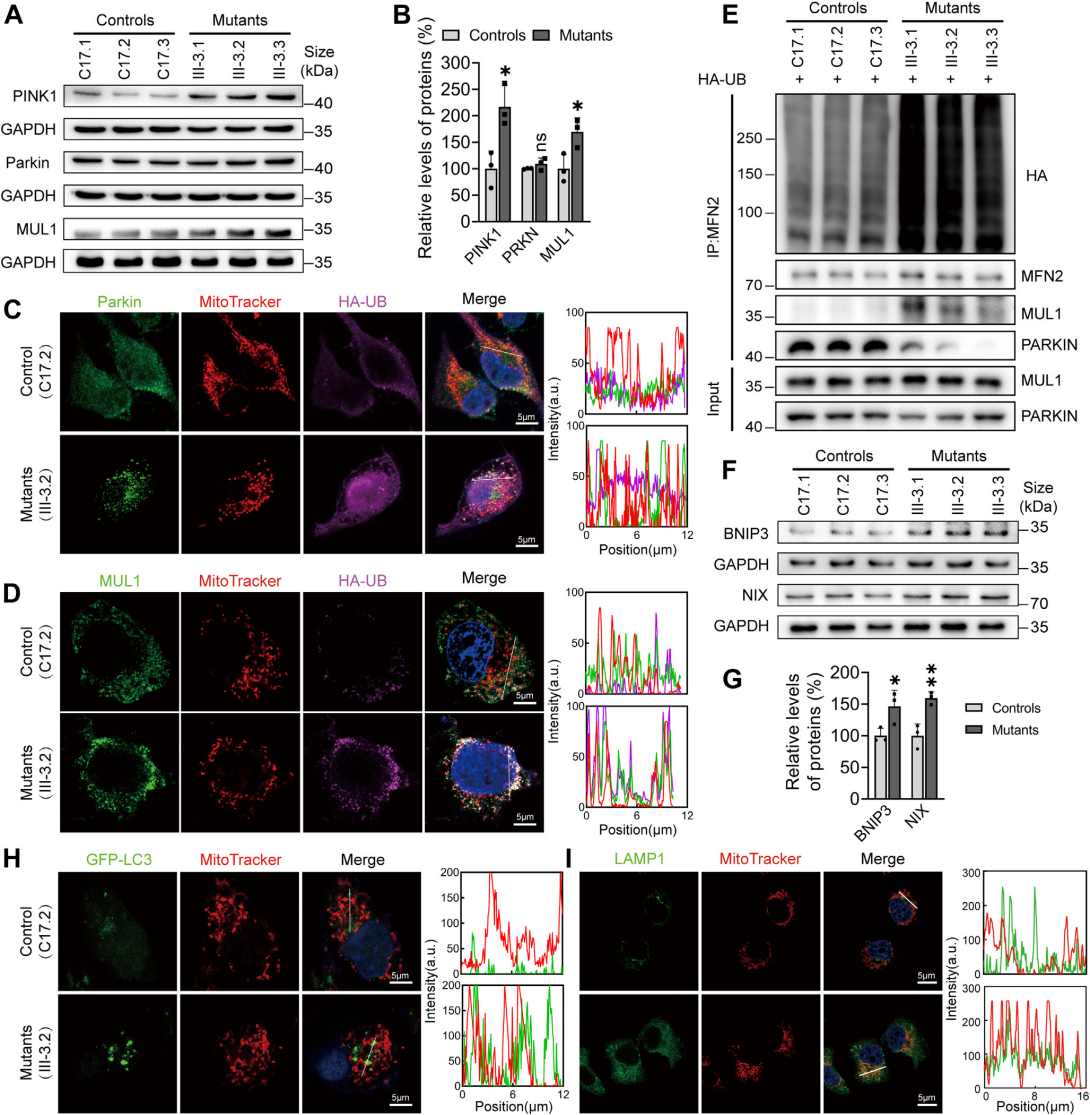
**Mitophagy assays**. *A*, Western blot analysis of ubiquitin-mediated mitophagy associated proteins (PINK1, Parkin and MUL1) in six cell lines with GAPDH as a loading control. *B*, quantification of ubiquitin-mediated mitophagy associated proteins. Three independent experiments were made for each cell line. *C*, *D*, immunofluorescence assays. The control (C17.2) and mutant (III-3.2) cybrids were transfected by HA-UB staining with MitoTracker and labeling with Parkin (*C*), or MUL1 (*D*) antibodies, conjugated to Alex Fluor 488 (*green*) analyzed by confocal microscopy, respectively. Scale bars: 5 μm. *E*, immunoprecipitation of MFN2 followed by immunoblotting for Parkin and MUL1 in the three control and three mutant cell lines expressing HA-UB. *F*, Western blot analysis of receptor-mediated mitophagy associated proteins (BNIP3 and NIX) in six cell lines with GAPDH as a loading control. *G*, quantification of receptor-mediated mitophagy associated proteins. Three independent experiments were made for each cell line. *H* and *I*, immunofluorescence analysis. The control (C17.2) and mutant (III-3.2) cybrids expressing GFP-LC3 (*H*), and endogenous expressing LAMP1 (*I*), staining with MitoTracker analyzed by confocal microscopy, respectively. Scale bars: 5 μm. Immunofluorescence colocalization analysis. Co-localization analysis of LAMP1 and mitochondria in control (C17.2) and mutant (III-3.2) cell lines.

Mitochondrial damage arising from mtDNA mutations mitigate mitophagy as another mitochondrial quality control mechanism to ameliorate organellar and cellular homeostasis (29, 36, 46, 47, 48). Mitophagy is a specific form of autophagy that identifies, removes, and replaces damaged mitochondria, ensuring optimal energy production and reducing oxidative stress (36, 48). Based on the targeting signals present on damaged or excess mitochondria that trigger the process, mitophagy can be classified as ubiquitin-dependent mitophagy, such as Parkin-dependent pathways, as well as ubiquitin-independent or receptor-mediated mitophagy, which involves apoptosis-related proteins acting as mitophagy receptors or inhibitors (48). In particular, MFN2 interacting with MUL1 acts as a substitute for PINK1/Parkin mitophagy (45, 49). The reduced levels of MFN2 indicated that the m.7516delA mutation affected the Parkin-dependent mitophagy pathway (PINK1-Parkin pathway), which is initiated by the recruitment of Parkin from the cytosol to the mitochondrial surface, ultimately resulting in mitophagy (48). As shown Figure 4, *A* and *B*, the mutant cybrids displayed increased levels of PINK1 but not Parkin. However, the mutant cybrids revealed marked increasing Parkin levels and ubiquitination than those in control cybrids (Fig. 4*C*). Elevating ubiquitin-dependent mitophagy was further supported by significant increasing levels of MUL1, which is mitochondrial ubiquitin E3 ligase, using MFN2 as ubiquitin substrate, and ubiquitination in the mutant cells (Fig. 4, *A* and *B*, *D*). The effects of m.7516delA mutation on MFN2-ubiquitination and interaction between MFN2 and MUL1 or Parkin were examined by the immunoprecipitation assays. As shown in Figure 4*E*, mutant cells exhibited marked increasing levels of MFN2-ubiquitination and elevated interaction between MFN2 and MUL1 but reduced interaction between MFN2 and Parkin, as compared with those in control cells. Furthermore, ubiquitination-independent mitophagy was regulated by pro-apoptotic proteins BNIP3 and NIX (36). As shown in Figure 4, *F* and *G*, the levels of BNIP3 and NIX in the mutant cybrids were significantly increased in the mutant cybrids as compared with control cybrids. The impact of m.7516delA mutation on mitophagy was further supported by markedly increased levels of LC3 and LAMP1 (lysosome-associated membrane glycoprotein1) in the mitochondria *via* the immunofluorescence assays using mutant and control cells transiently expressing GFP-LC3 or staining with LAMP1 antibody (Fig. 4, *H* and *I*). These results indicated that the m.7516delA mutation upregulated both ubiquitin-dependent and ubiquitin-independent mitophagy pathways.

### Activation of ISR arising from m.7516delA mutation-induced deficiency

**Figure 5:**
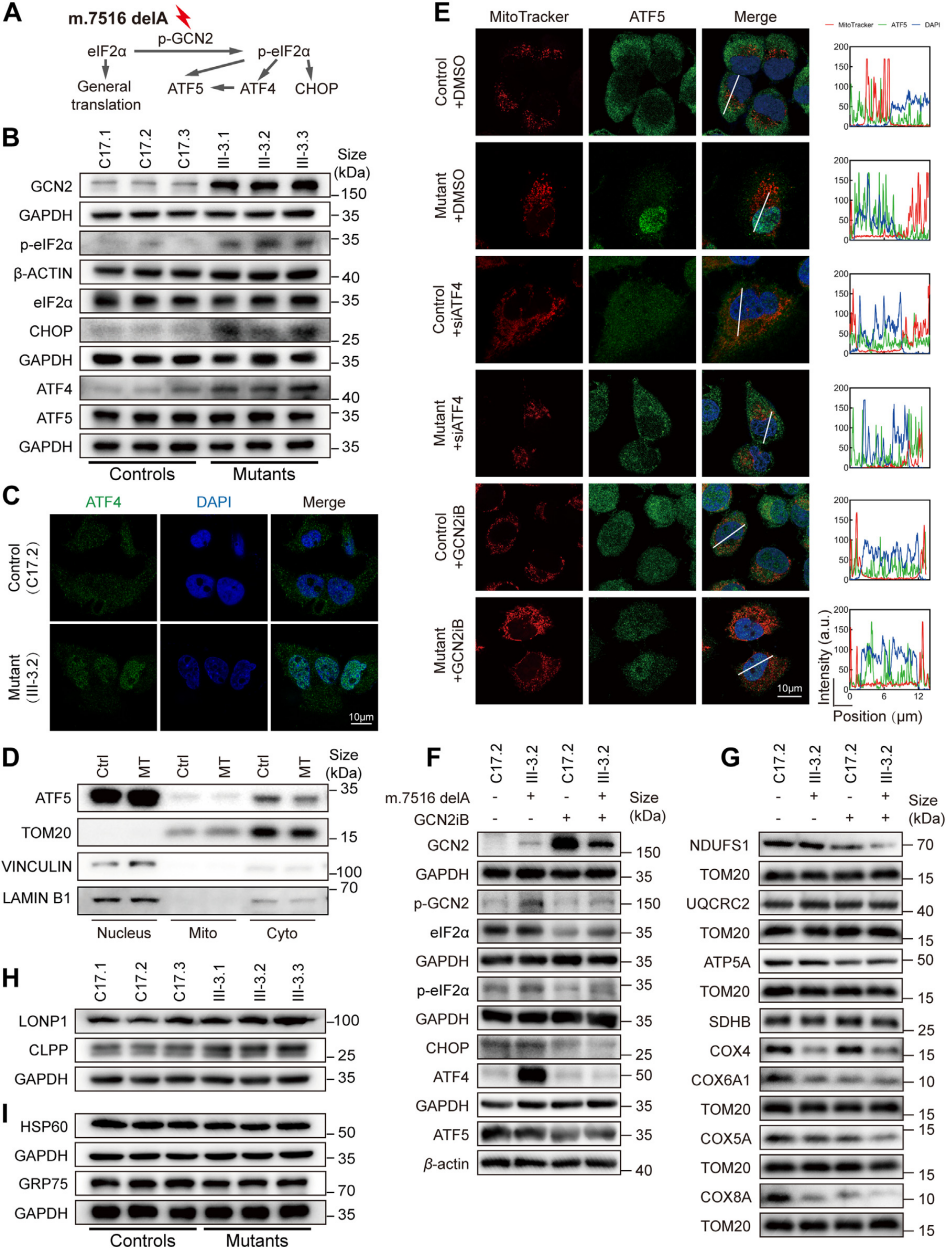
**Assessment of UPR^mt^ activation**. *A*, the UPR^mt^ activation in response to mitochondrial dysfunctions. Mitochondrial dysfunctions induce the phosphorylation of kinase GCN2, which phosphorylates the translation initiation factor eIF2α. eIF2α phosphorylation results in generally reduced protein synthesis with a concomitant increase in translation of those mRNAs encoding CHOP, ATF4 and ATF5. These induced the expression of mitochondrial proteases and chaperones to repair mitochondrial damages (4). *B*, Western blot analysis of UPR^mt^ related proteins: GCN2, p-eIF2α, eIF2α, CHOP, ATF4 and ATF5 among six cell lines. *C*, immunofluorescence analysis. The distributions of ATF4 from cybrids (C17.2 and III.3–2) were visualized by immunofluorescent labeling with ATF4 antibody conjugated to Alex Fluor 488 (*green*) analyzed by confocal microscopy. DAPI-stained nuclei were shown by fluorescence (*blue*). Scale bars: 10 μm. *D*, the levels of ATF5 in nucleus, cytosol and mitochondria in mutant and control cell lines were measured by fractioning the cells into nuclear, mitochondrial and cytosolic fractions and Western blot analysis using antibodies: ATF5, Tom20, Vinculin and Lamin B1 for mitochondrial, cytosolic and nuclear proteins, respectively. Nucleus; Cyto, cytosol; Mito, mitochondria. *E*, immunofluorescent staining with mitochondrial dye MitoTracker (*red*) and labeling with ATF5 antibody conjugated to Alex Fluor 488 (*green*) analyzed by confocal microscopy in control and mutant cybrids after siATF4 transfection and 1 μM GCN2iB or DMSO treatment. Scale bars: 10 μm. *F* and *G*, Western blot analysis of proteins involved in GCN signaling and OXPHOS in control (C17.2) and mutant (III-3-2) cybrids after 1 μM GCN2iB or DMSO treatment: (*F*) GCN signaling-associated proteins (GCN2, p-eIF2α, eIF2α, CHOP, ATF4 and ATF5), (*G*) OXPHOS associated proteins (NDUFS1, SDHD, UQCRC2, ATP5A, COX4, COX5A, COX6A1, COX8A). *H* and *I*, Western blot analysis of mitochondrial proteostasis LONP1 and CLPP (*H*) and mitochondrial chaperones HSP60 and GRP75 (*I*) among six cell lines.

We investigated cellular responses to the m.7516delA mutation-induced dysfunctions to maintain organellar and cellular integrity through ISR pathway, including UPR^mt^ and UPR^ER^ (30, 31, 32, 33, 34). As shown in Figure 5*A*, ISR pathway promotes the transcription of various transcription factors, including ATF4, ATF5, and CHOP, along with other nuclear-encoded components such as chaperones and proteases, to facilitate the repair of mitochondrial damage caused by mitochondrial DNA mutation through the phosphorylation of GCN2 (30, 31, 32). Here, we performed a Western blot analysis to measure the levels of ISR-related proteins, mitochondrial proteases and chaperones in mutant and control cybrids. As shown in Figures 5, *B* and *C*, *F* and S2*A*, the mutant cybrids revealed markedly increased levels of GCN2, P-GCN2, p-eIF2α, CHOP, and ATF4, but no significant changes in levels of ATF5 and eIF2α responsible for general translation, as compared with those in control cybrids, indicating the activation of ISR pathway. The effects of the m.7516delA mutation on subcellular localization of ATF4 and ATF5 were further examined by immunofluorescence and Western blot assays. As shown in Figure 5*C*, mutant cybrids displayed notable increases in the levels of ATF4 in the nucleus, when compared to control cybrids. The levels of ATF5 in the nucleus were further assessed by separating the cells into nuclear, cytosolic, and mitochondrial fractions, followed by Western blot analysis. As shown in Figure 5*D* and supplemental 2B, the mutant cybrids exhibited elevated levels of ATF5 in the nucleus but decreased levels in the cytosolic fractions, compared to control cybrids. To further assess if the nuclear translocation of ATF5 depends on ATF4 and/or p-eIF2α, we generated the mutant and control cybrids with reduced expressions of ATF4 and p-eIF2α by knockdown or treatment with GCN2iB and analyzed the subcellular localization of ATF5. As shown in Figure 5*E*, knockdown ATF4 resulted in drastic decreasing nuclear translocation of ATF5 in mutant cybrids but mild elevation in the nuclear translocation of ATF5 control cybrids, while the inhibited phosphorylation of eIF2α with treatment of GCN2iB gave rise to similar consequence to ATF4-knockdown cells (Fig. 5*F*).

The activation of ISR pathway was further assessed by treating mutant and control cybrids with GCN2iB, an ATP-competitive inhibitor of GCN2 (50). After the administration of 2 mM GCN2iB for 12 h, elevating levels of GCN2 in the control cybrids were much higher than those in mutant cybrids, whereas drastic decreases in the levels of P-GCN2 occurred in mutant cybrids, when compared with control cybrids (Figs. 5*F* and S2*C*). Furthermore, the expression of CHOP and ATF4 in the presence of GCN2iB was pronouncedly decreased in the mutant cybrids, as compared with those in control cybrids. Strikingly, the administration of GCN2iB led to much lower ratios of p-eIF2α/eIF2α in the mutant cybrids than those in control cybrids. However, the treatment of GCN2iB reduced the similar levels of ATF5 between control and mutant cybrids. To investigate whether the activation of ISR contributed to the lower expression of nucleus-encoding OXPHOS subunits, we measured the levels of NDUFS1, SDHB, UQCRC2, COX4, COX5A, COX6A1, COX8A, and ATP5A in the mutant and control cybrids in the presence and absence of GCN2iB to reduce GCN2 activity (50). A shown in Figures 5*G* and S2*D*, the treatment of GCN2iB resulted in reduced levels of COX5A, COX8A, and COX6A1 but no significant change in the levels of SDHB, UQCRC2, COX4, and ATP5A, respectively.

We then evaluated whether the m.7516delA mutation-induced deficiency regulated the expression of genes involved in chaperones and proteases. As illustrated in Figures 5*H* and 2*E*, these mutant cybrids revealed a slight increase in the levels of LONP1, which plays a role in maintaining mitochondrial proteostasis, and CLPP, which is involved in mitochondrial ribosome assembly (51, 52). However, there are no significant differences in the levels of mitochondrial chaperones HSP60 and GRP75 between mutant and control cybrids (Figs. 5*I* and S2*F*).

We further assessed whether the m.7516delA mutation affected the UPR^ER^ pathway which is a cellular stress response related to the endoplasmic reticulum (ER) stress. As shown in the supplemental 3A and 3B, there were no significant differences in the levels of UPR^ER^-related proteins, including IRE1A, P-IRE1A, PERK, and GRP78 (Bip) (39), between the mutant and control cybrids. The effect of m.7516delA on the UPR^ER^ pathway was further assessed by treating mutant and control cybrids with thapsigargin to induce ER stress (53). As illustrated in Figures S3*C* and S3*D*, the ratios of p-PERK/PERK in mutant cybrids in the presence of thapsigargin were increased as much as those in control cybrids, while the presence and absence of thapsigargin did not change significantly the level of GRP78 in the mutant and control cybrids. Furthermore, the administration of thapsigargin caused the elevation of ATF4 in control cybrids but did not change the levels of ATF4 in the mutant cybrids, indicating the increasing level of ATF4 in the mutant cells induced by UPR^mt^ but not UPR^ER^. These data indicated that the deficiency induced by the m.7516delA mutation activated the ISR through UPR^mt^ rather than proteostasis stress and the UPR^ER^ pathway.

### Upregulated autophagy

**Figure 6:**
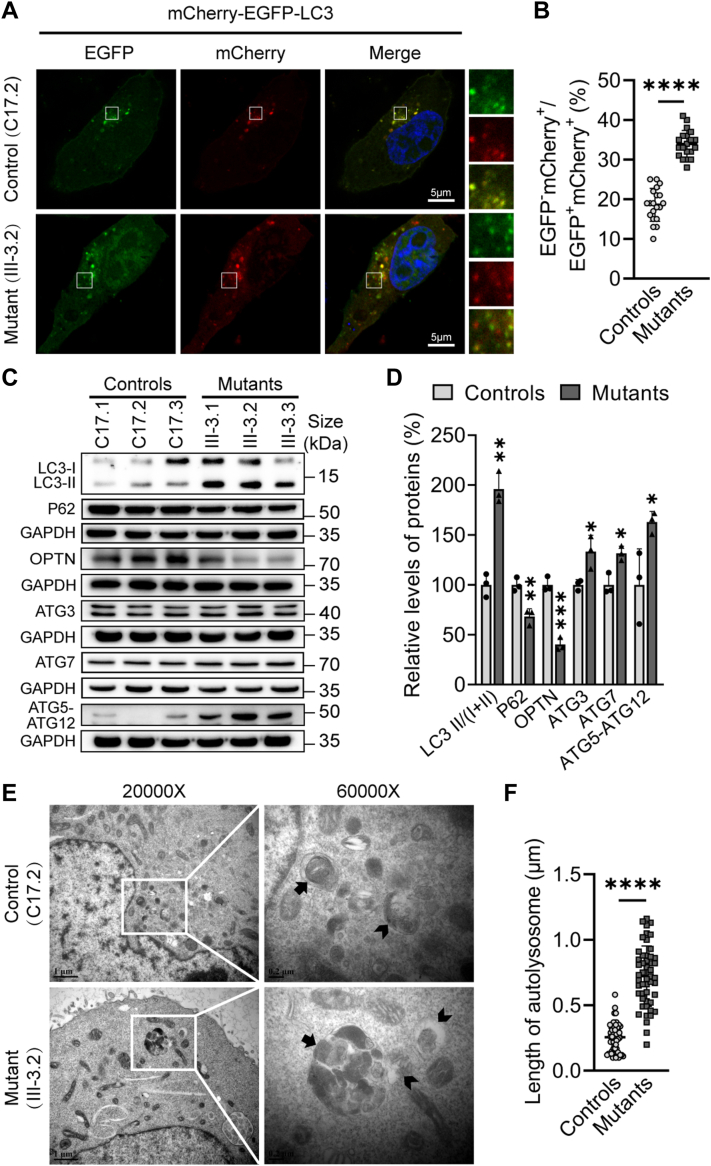
**Analysis of autophagy**. *A*, immunofluorescence assays. Control cybirds (C17.2) and mutant cybrids (III-3.2) expressing the mCherry-EGFP-LC3 were imaged by confocal microscopy. Scale bars: 5 μm. *B*, measurement of the autophagy activity. The mCherry-positive but EGFP-negative puncta were quantified for each cell lines (n = 20). *C*, Western blot analysis of 6 autophagy-associated proteins among six cell lines with GAPDH as a loading control. *D*, quantification of markers of 6 autophagy-associated proteins. Three independent determinations were done in each cell line. *E*, cells from mutant and control cybrids were examined by transmission electron microscopy of initial autophagic vacuoles (*arrow*), degradative autophagic vacuoles (arrow with tail). Ultrathin sections were stained with uranyl acetate and alkaline lead citrate. 20,000 and 60,000 × magnifications were used, respectively. *F*, quantification of the length of autolysosomes (n = 50). Graph details and symbols are explained in the legend to Figure 1*F*.

We investigated the cellular response to mitochondrial dysfunctions arising the m.7516delA mutation to reshape cellular homeostasis through autophagy (28, 29, 46, 54). The effects of m.7516delA mutation-induced deficiencies on autophagy were assessed using immunoblotting and immunofluorescence assays including the mCherry-EGFP-LC3 reporter system. In this system, GFP fluorescence is rapidly quenched in the acidic lysosomal environment, whereas mCherry fluorescence remains. Yellow (EGFP^+^ and mCherry^+^) LC3 puncta represented autophagosomes, while red LC3 puncta (EGFP^−^ and mCherry^+^) indicated autolysosomes. As shown in Figure 6, *A* and *B*, mutant cybrids displayed markedly increases in the proportion of red puncta, as compared with control cybrids, indicating that the m.7516delA mutation facilitated autophagy. The effect of m.7516delA mutation on autophagy was further assessed using Western blot analysis with the following markers: LC3, P62, and OPTN involved in the initiation phase, ATG3, ATG7, and ATG12 involved in autophagosome formation and maturation (36, 54). As shown in Figure 6, *C* and *D*, the average levels of LC3 II/(I + II), P62, OPTN, ATG3, ATG7, and ATG5-ATG12 in the three mutant cell lines were 196%, 68%, 40%, 133%, 131%, and 163% of mean values measured in three control cell lines, respectively. To further evaluate the effect of m.7516delA mutation on autophagy, we examined autophagy process and quantified autophagic accumulation in mutant and control cells using transmission electron microscopy. As shown in Figure 6, *E* and *F*, mutant cells exhibited a predominance of enlarged mature late autophagic vacuoles compared to early autophagic vacuoles, which contained morphologically intact cytoplasm, in contrast to control cells. These data suggest that the m.7516delA mutation upregulated autophagy pathway.

### Promoted intrinsic apoptosis

**Figure 7:**
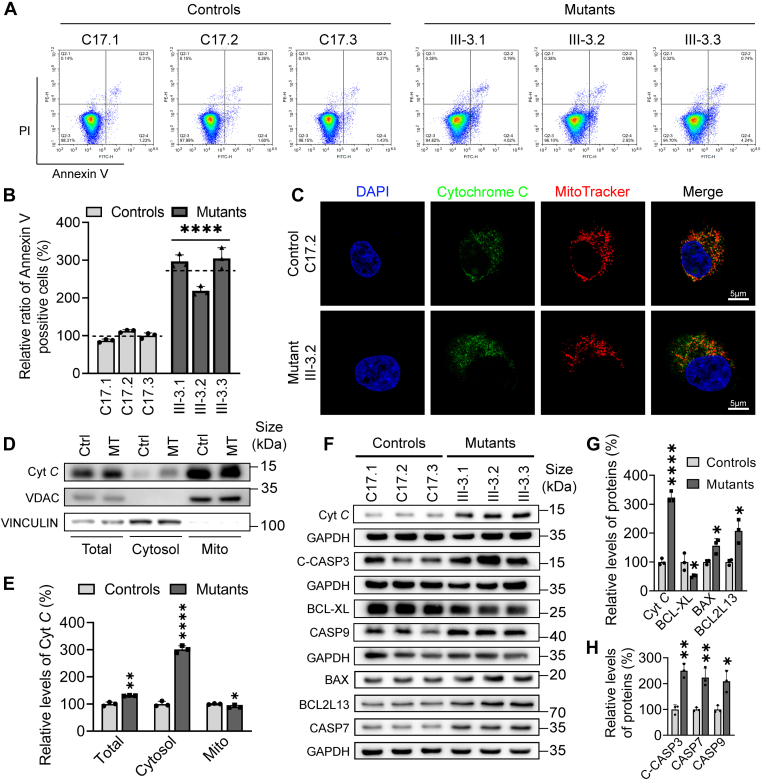
**Apoptosis assays**. *A*, annexin V/PI apoptosis assay by flow cytometry among six cell lines. Cells were harvested and stained with Annexin V and 1 μl of propidium iodide. The percentages of Annexin V-positive cells were assessed. *B*, annexin V-positive cells from various cell lines. Three independent determinations were done in each cell line. *C*, immunofluorescence analysis. The distributions of cytochrome c from mutant III-3.2 and control C17.2 cybrids were visualized by immunofluorescent staining with mitochondrial dye MitoTracker and labeling with cytochrome c antibody conjugated to Alex Fluor 488 (*green*) analyzed by confocal microscopy. DAPI stained nuclei were identified by their *blue* fluorescence. Scale bars: 5 μm. *D*, the levels of cytochrome c in cytosol in mutant and control cell lines were measured by fractioning the cells into mitochondrial and cytosolic fractions and Western blot analysis using antibodies of cytochrome c, VDAC for mitochondrial protein and Vinculin for cytosolic protein. Total, total cell lysate; Cytosol, cytosol; Mito, mitochondria. *E*, quantification of cytochrome c levels among total cell lysate, cytosol and mitochondria fraction of mutant cybrids (III-3.2) and control cybrids (C17.2). Three independent determinations were made on each cell line. *F*, Western blot analysis of 6 apoptosis-associated proteins. Total cellular proteins (20 μg) from various cell lines were electrophoresed, electroblotted and hybridized with seven apoptosis-associated protein antibodies: cytochrome C, BCL-X_L_, BCL2L13, BAX, uncleaved caspase 7 and caspase 9, cleaved caspase 3, with GAPDH as a loading control. *G* and *H*, quantification of apoptosis-associated proteins: cytochrome c, BCL-X_L_, BCL2L13, BAX, uncleaved caspase 7 and caspase 9, cleaved caspase 3. Three independent experiments were made for each cell line. Graph details and symbols are explained in the legend to Figure 1.

We the assessed whether the m.7516delA mutation-induced deficiencies alleviate cellular integrity *via* the process of intrinsic apoptosis. The effect of m.7516delA mutation on apoptotic process was examined by Annexin V/PI-based flow cytometry for cellular apoptosis, immunofluorescence and Western blot assays. As shown in Figure 7, *A* and *B*, the average ratios of Annexin V-positive cells in the mutant cell lines carrying the m.7516delA mutation were 274% of the mean values measured in the control cell lines. As shown in Figure 7*C*, the immunofluorescence patterns of double-labeled cells with rabbit monoclonal antibody specific for cytochrome c and MitoTracker revealed significantly increased levels of cytochrome c in the mutant cells, compared with control cells. The levels of cytochrome c in cytosol in mutant and control cell lines were further evaluated by fractioning the cells into mitochondrial and cytosolic fractions and Western blot analysis. As shown in Figure 7, *D* and *E*, *F* and *G*, the levels of cytochrome c in the mutant cell lines were markedly increased in the total cellular and cytosolic fractions, but not in mitochondrial fraction, when compared to those in control cell lines. Furthermore, we examined the levels of one apoptosis inhibited protein (BCL-X_L_) and 5 activated proteins (BAD, BCL2L 13, caspases 3, 7, and 9) in mutant and control cell lines by Western blot analysis (55, 56). As shown in the Figure 7, *F* and *G*, *H*, the average levels of BCL-X_L_, BAD, BCL2L13, caspases 3, 7, and 9 in three mutant cell lines were 51%, 156%, 207%, 142%, 223%, and 209% of mean values measured in three control cell lines, respectively. These results suggested that the m.7516delA mutation promoted the intrinsic apoptotic process.

## Discussion

In this study, we investigated the mechanism underlying mitochondrial tRNA processing defects arising from the deafness-associated m.7516delA mutation reprogramed mitochondrial and cellular homeostasis *via* activating both ISR and PINK1/Parkin mitophagy pathways. In the previous investigation, we demonstrated that the m.7516delA mutation caused asymmetrical defects in the processing of tRNAs in the heavy-strand and light-strand polycistronic transcripts and resultant aberrant RNA metabolisms impaired the synthesis of mtDNA-encoded ND4, ND5, CO1, CO2, and CO3, subunits of OXPHOS (21). These mtDNA-encoded subunits function as precursors for the formation of new complexes, which necessitate the import and assembly of nucleus-encoded subunits with the help of assembly factors for OXPHOS complexes (40, 57). Thus, impaired synthesis of these mtDNA encoded polypeptides arising from the m.7516delA mutation dysregulated the expression of nucleus-encoded subunits and assemble factors of OXPHOS complexes and contributed to the downregulation of complex IV subunits (COX5A, COX4, COX8A, and COX6A1), upregulation of assemble factors FOXRED1 and COX16, but no effect of other nucleus-encoding subunits of OXPHOS complexes and their assemble factors. Alternatively, the activation of ISR also exerted the expression of nucleus-encoding components of OXPHOS complexes, evidenced by reduced levels of NDUFS1, COX5A, COX8A, and COX6A1 with treatment of GCN2iB (50). Thus, both impaired synthesis of mtDNA-encoding ND4, ND5, CO1, CO2, and CO3 and lower levels of nucleus-encoding COX5A, COX4, COX8A and COX6A1 produced aberrant assembly, reduced activities of complexes I and IV rather than complexes II, III and V. The resultant respiratory deficiencies caused diminished mitochondrial ATP production and membrane potential, increased production of reactive oxygen species and subsequent failure of cellular energetic processes required for hearing function.

To respond to the m.7516delA mutation-induced dysfunction, the mitochondria may reprogram their integrity to regulate their quality control *via* fusion and fission processes as well as mitophagy which initiates the breakdown of severely damaged mitochondria (35, 36, 37, 38). The effect of mitochondrial quality control by the m.7516delA mutation was evidenced by the abnormality of mitochondrial morphologies, characterized by increasing fragmented mitochondria and decreasing elongated mitochondria in the mutant cells, suggesting the imbalance between mitochondrial fission and fusion. These fragmental mitochondria are likely to be targeted for mitophagy. Especially, the m.7516delA mutation promoted mitochondrial fission, as evidenced by upregulating fission-related genes, including DRP1, FIS1, and MFF, compared to control cells without the mutation. Conversely, significant decreases in the levels of fusion-related protein MFN2, but not OPA1 or MFN1, were noted in cells carrying the m.7516delA mutation. The decreased level of MFN2 suggested the impact of m.7516delA mutation on Parkin dependent mitophagy (PINK1-Parkin Pathway) as MFN2 interacted with MUL1 acts as a substitute for PINK1-Parkin-mediated mitophagy (45, 49). In this study, we demonstrated that the cell bearing the m.7516delA mutation exhibited increasing Parkin level, MFN2-ubiquitination and altering interaction between MFN2 and MUL1 or Parkin, indicating upregulation of the ubiquitin-dependent mitophagy. Furthermore, ubiquitination-independent mitophagy was also upregulated by the m.7516delA mutation-induced ISR pathway supported by elevated expression of BNIP3L and NIX (47, 48). The effect of m.7516delA mutation on mitophagy was further supported by markedly increased levels of LC3 and LAMP1. These findings suggested that the m.7516delA mutation upregulated both ubiquitination-dependent and ubiquitination-independent mitophagy pathways. These results were consistent with those in cells carrying the deafness-associated 12S rRNA 1555A>G, tRNA^Phe^ 593T>C or LHON-associated tRNA^Ala^ 5587T>C mutations (28, 29, 58) and but contrast with the upregulation of ubiquitination-dependent mitophagy but downregulation of ubiquitination-independent mitophagy in the cells carrying the LHON-associated ND1 3460G>A or ND6 14484T>C mutation (59, 60). These discrepancies may reflect the impaired synthesis of multiple polypeptides due to the 12S rRNA and tRNA mutations but only one defective polypeptide due to ND1 3460G>A or ND6 14484T>C mutation. This interplay between mitochondrial dynamics and mitophagy assures the homeostasis of cells.

Strikingly, the m.7516delA mutation-induced mitochondrial dysfunctions activated ISR pathway to act as a repair-oriented pathway that minimizes damage in defective mitochondria and promotes the restoration of salvageable organelles. In fact, the activation of ISR required the phosphorylation of eIF2α, catalyzed by one of four kinases (GCN2 (amino acid starvation), PERK (ER stress), PKR (presence of double-stranded RNA), and HRI (heme deficiency)) (34, 61). In this study, we demonstrated that the m.7516delA mutation activated ISR pathway, evidenced by upregulation of GCN2, P-GCN2, p-eIF2α, CHOP, and ATF4 in the mutant cybrids. The activated ISR pathway was further supported by elevated levels of ATF5 in the nucleus but decreased levels in the cytosolic fractions and drastic decreasing nuclear translocation of ATF5 in the knockdown ATF4 in the mutant cybrids. Furthermore, the administration of GCN2iB led to less increased levels of GCN2, drastic decreases in the levels of P-GCN2, CHOP and ATF4, and much lower ratios of p-eIF2α/eIF2α in the mutant cybrids than and those in control cybrids. Here, the upregulation of GCN2 arising from the m.7516delA mutation-induced dysfunctions apparently accounted for marked increases in the abundance of phosphorylated eIF2α, promoted ATF4 activation, raised the expression of CHOP and triggered the activation of ISR pathway (62). Stimulation of phosphorylated eIF2α by the m.7516delA mutation resulted in reduced global translation alongside the selective synthesis of proteins, including nucleus-encoding subunits and assemble factors of OXPHOS complexes (62). In particular, the activated ISR pathway due to the m.7516delA mutation specifically contributed to lowered expression of nucleus-encoded complex IV subunits, supported by the evidences that the treatment of GCN2iB led to reduced levels of COX5A, COX8A, and COX6A1 but no significant change in the levels of SDHB, UQCRC2, COX4, and ATP5A. However, the m.7516delA mutation only caused mild increases in the mitochondrial proteases (LONP1 and CLPP) but did not elevate the expression of mitochondrial chaperones (HSP60 and GRP75) (51, 52). These observations were in contrast with the ISR pathway activation due to other mitochondrial stressors (34, 61, 62, 63). Furthermore, these mitochondrial dysfunctions did not activate UPR^ER^ pathways, evidenced by no change in the levels of UPR^ER^-related proteins IRE1A, P-IRE1A, PERK, and GRP78 (Bip) (39). Especially, the administration of thapsigargin caused the elevation of ATF4 in control cybrids but did not change the levels of ATF4 in the mutant cybrids, indicating the increasing level of ATF4 in the mutant cells induced by UPR^mt^ but not UPR^ER^. Therefore, the activation of ISR pathway plays an important role in restoring mitochondrial function and maintaining cellular homeostasis in response to mitochondrial stressors arising from the m.7516delA mutation.

Both activation of ISR and PINK1/Parkin mitophagy pathways contributed to maintaining the cell homeostasis *via* regulating autophagy and apoptosis. Autophagy, a lysosome-dependent degradation process, serves as a vital mechanism for adapting to stress and ensuring intracellular quality control (54). In this study, the upregulation of the autophagy occurred in the cells harboring the m.7516delA mutation, evidenced by significant accumulations of matured late autophagic vacuoles and marked increases in LAMP1 and LC3 levels. The upregulation of autophagy process was further supported by decreased levels of P62 and OPTN (initiation phase), increased levels of ATG3, ATG7, and ATG5-ATG12 (formation and maturation of autophagosomes) in mutant cells carrying the m.7516delA mutation. Upregulated autophagy facilitated the breakdown and recycling of aged cellular components in the mutant cybrids harboring the m.7516delA mutation, as in the cases of these cells harboring the mt-tRNA^Met^ 4435A>G, mt-tRNA^Ile^ 4295A>G, and mt-tRNA^Ala^ 5587T>C mutations (25, 26, 46). Alternatively, the m.7516delA mutation elevated intrinsic apoptotic process to facilitate the removal of damaged cells. In particular, the mutant cybrids bearing the m.7516delA mutation exhibited markedly elevated Annexin V intensity and release of cytochrome *c* into the cytosol. The release of cytochrome facilitated the activation of cleaved-caspase 3, caspase 7, and caspase 9, thereby initiating cell death (56). The upregulation of intrinsic apoptotic process was further supported by increased levels of BAX and BCL2L13 and decreased levels of anti-apoptotic proteins such as BCL-X_L_ in the cybrids carrying the m.7516delA mutation. This suggested that the m.7516delA mutation-induced activation of both ISR and PINK1/Parkin mitophagy pathways reprogrammed cell homeostasis by upregulating autophagy and apoptosis.

In summary, we demonstrated the impact of deafness associated with the m.7516delA mutation on organellar and cellular homeostasis contributed to the pathological process of hearing loss. The m.7516delA mutation dysregulated the expression of nucleus-encoding subunits and assembly factors of OXPHOS complexes and altered the assembly and activities of OXPHOS complexes. The m.7516delA mutation-induced deficiencies regulated mitochondrial integrity *via* activation of ISR and PINK1/Parkin mitophagy pathways and by causing mitochondrial dynamic imbalance towards fission. Finally, the m.7516delA mutation-induced deficiencies reprogrammed the cell homeostasis of cells *via* promoting autophagy and intrinsic apoptosis. The broad effects of the m.7516delA mutation on mitochondrial and cellular integrity impact various aspects of hearing function, thereby being integral to the pathogenesis of deafness. Thus, our findings may provide new insights into pathogenic mechanism of maternally inherited deafness arising from reprogramming mitochondrial and cellular homeostasis due to mitochondrial tRNA processing defects.

## Experimental procedures

### Cell lines and culture conditions

Three mutant cybrids (III-3.1, III-3.2, and III-3.3) bearing the homoplasmic m.7516delA mutation derived from a Han Chinese family with maternal inheritance of deafness and three control cybrids (C17.1, C17.2, and C17.3) derived from normal hearing Chinese control subject lacking the mutation but belonging to the same mtDNA haplogroup were generated as described previously (21). All cell lines were grown in Dulbecco’s Modified Eagle Medium (DMEM) (Gibco) (containing 4.5 mg of glucose, 0.11 mg pyruvate/ml and 50 μg of uridine/ml), supplemented with 10% fetal bovine serum (FBS) (Gibco).

### Western blot analysis

Western blot analysis was undertaken as described elsewhere (22). Briefly, 20 μg of total cellular proteins obtained from various cell lines were denatured and electrophoresed through 10% bis-Tris SDS-polyacrylamide gels. Afterward, the gels were electroblotted onto polyvinylidene difluoride (PVDF) membrane for hybridization. Membranes were blocked in Tris-Buffered Saline and Tween20 (TBST) (150 mM NaCl, 10 mM Tris–HCl, pH 7.5 and 0.1% Tween 20) containing 5% (w/v) milk, then incubated with the corresponding primary and secondary antibodies. The primary antibodies used for this investigation were as described in the Table S1. Peroxidase Affinipure goat anti-mouse IgG and goat anti-rabbit IgG (Beyotime) were used as secondary antibodies, and protein signals were detected using the ECL system (CWBIO).

### Blue native polyacrylamide gel electrophoresis (BN-PAGE) and in-gel activity assays

BN-PAGE was performed on mitochondrial protein extracted from various cell lines as detailed elsewhere (41, 42). For in-gel activity assays, samples containing 30 μg of total mitochondrial proteins were separated on 3% to 12% Bis-Tris Native PAGE gel. The native gels were prewashed in cold water and then incubated with the specific substrates of OXPHOS [NADH and NTB for complex I, sodium succinate, phenazine methosulfate, and NTB for complex II, DAB and cytochrome c for complex IV, glycine, MgSO_4_, ATP and Pb (NO_3_)_2_ for complex V] at room temperature (42). After stopping reaction with 10% acetic acid, gels were washed with water and scanned to visualize the activities of respiratory chain complexes.

### Immunofluorescence analysis

Immunofluorescence experiments were undertaken as described elsewhere (46, 64, 65). Cells were cultured on cover glass slips (Thermofisher), fixed in 4% formaldehyde for 15 min, permeabilized with 0.2% Triton X-100, blocked with 5% FBS for 1 h, and immune-stained with DRP1, Cytochrome *c*, LC3B, LAMP1, Parkin, and BNIP3L antibodies overnight at 4 °C, respectively. The cells were then incubated with Alex Fluor 594 goat anti-rabbit IgG, Alex Fluor 488 goat anti-rabbit IgG and Alex Fluor 488 goat anti-mouse IgG (Thermofisher), stained with 4′, 6-diamidino-2-phenylindole (DAPI) (Thermofisher) for 15 min and mounted with Fluoromount (Sigma-Aldrich). Cells were examined using a confocal fluorescence microscope (Olympus Fluoview FV1000) with three lasers (Ex/Em = 550/570, 492/520, and 358/461 nm). The fluorescence intensity was quantified using ImageJ software, and the data were further analyzed by Microsoft Excel and GraphPad Prism 9.

### Gene expression analysis

Total cellular RNAs were obtained from various cell lines using TRIzol reagent (Invitrogen, 15,596,026) and reverse transcribed into cDNA using PrimeScript RT Master Mix (Takara, RR036 A). RT-qPCR was performed on the Applied Biosystems 7900HT Fast Real-Time PCR System. These data were analyzed using the 7900 System SDSRQ Manager and relative gene expressions were calculated using the 2^-ΔΔCt^ method using GAPDH as a housekeeping gene. The sequences of primers are as follows:

COX4, F, 5′-TGGCGGCAGGTGTACATTTT-3′;

COX4, R, 5′-AGTCTTCGCTCTTCACAACACT-3′;

COX5A, F, 5′-GGCTTAGGGGACTGGTTGTC-3′;

COX5A, R, 5′-CCGTAAGAGGGCTTGGCTAC-3′;

COX6A1, F, 5′-AATGGCGGTAGTTGGTGTGT-3′;

COX6A1, R, 5′-GTGGTGCGACTTCAGGTACA-3′;

COX8A, F, 5′-GAATTGGCCGTTGGGCTTAC-3′;

COX8A, R, 5′-CAGAACGGACCCCTTCACTC-3′;

COX16, F, 5′-AGTTCTGAGCCGATGGAAGAG-3′;

COX16, R, 5′-ACGAAGACCAAAAGAACCTCCA-3′.

### Transmission electron microscopy

The cell lines were washed with PBS and fixed in 2.5% glutaraldehyde in phosphate buffer (0.1 M, pH 7.0) for 4 h and post-fixed with 1% OsO_4_ in phosphate buffer (0.1 M, pH 7.0) for 2 h. The samples were dehydrated with increasing concentrations of ethanol (30, 50, 70, 80, 90, 95, and 100%) and transferred to absolute acetone for 20 min. After placing in a 1:1 mixture of absolute acetone and the final Spurr resin (SPI-CHEM, 02690-AB) mixture for 1 h at room temperature, the samples were transferred to a 1:3 mixture of absolute acetone and the final resin mixture for 3 h and to the final Spurr resin mixture overnight. Electron photomicrographs were taken from the ultrastructure of cells under a transmission electron microscope (Hitachi, H-7650).

### Cell extracts and subcellular fractionations

The control and mutant cell lines were washed once with 4 °C PBS and incubated with IP lysis buffer containing 1% EDTA-free protease inhibitor cocktail for 30 min at 4 °C. Whole-cell lysates were centrifuged at 17,000×*g* for 15 min at 4 °C to separate soluble proteins from cell debris. Subcellular fractions were prepared from cells using the cell fractionation kit (Abcam) in the presence of 1% EDTA-free protease inhibitor cocktail.

### Plasmid transfection

Cells were allowed to attach overnight in 24-well plates at a density of 1 × 10^5^ cells each well. The following day, HA-UB, GFP-LC3, or mCherry-EGFP-LC3 were transfected into cybrids using Hieff Liposomal Transfection Reagent (Yeasen) according to the manufacturer’s instructions. The transfection medium was replaced with fresh complete medium after 24 h to reduce cytotoxicity. The cells were transfected for 24 h before immunofluorescence analysis (46).

### Immunoprecipitation assay

The control and mutant cybrids (2.0 × 10^6^) were transfected with 12 μg of pcDNA3.1-UB-HA for 36 h using Hieff Trans liposomal transfection reagent following the manufacturer’s protocol (Yeasen), as detailed elsewhere (64). The cells were harvested and suspended with 1 ml of lysis buffer [1% NP40; 50 mM Tris–HCl, pH 7.6; 150 mM NaCl; 1 × Protease Inhibitor Cocktail (Bimake)] and lysed on ice for 30 min. The lysates were centrifuged at 20,000 g for 10 min at 4 °C. The supernatants were incubated with 40 μl of beads (cross-linked to 25 μg of MFN2 antibody) overnight at 4 °C with rotation. Beads were washed 4 times, then boiled for 5 min after SDS loading was added. Finally, the IP fractions were analyzed by Western blot analysis.

### siRNA transfection

siRNA transfections were performed with Hieff Trans *in vitro* siRNA/miRNA Transfection Reagent according to the manufacturer’s instructions. Cells were cultured in a six-well plate, and siRNAs were used at a concentration of 20 nM. Cells were collected at 24 h after transfection for immunofluorescence analysis. siRNAs targeting ATF4 used in this study were synthesized by RiboBio. The sequences used for siRNA-ATF4 were as follows: 5′-GCGUUGCUGUAACCGACAA-3’.

### Annexin V/PI apoptosis assay by flow cytometry

For discrimination of apoptotic and non-apoptotic cells by Annexin V/PI staining, cells were harvested and stained with Annexin V and 1 μl of propidium iodide (PI) (Beyotime) according to the manufacturer’s instructions. Each sample was detected by NovoCyte (ACEA Biosciences) and analyzed using NovoExpress software (58).

### Statistical analysis

All statistical analyses were performed using GraphPad Prism (version 8.00) for statistical analysis to compare outcomes using a two-tailed unpaired Student’s *t* test. *P* values of less than 0.05 were considered to be statistically significant. ∗*p* < 0.05; ∗∗*p* < 0.01; ∗∗∗*p* < 0.001; ∗∗∗∗*p* < 0.0001; ns, not significant.

## Data availability

The authors declare that all relevant data of this study are available within the article or from the corresponding author (gminxin88@zju.edu.cn) upon reasonable request.

## Supporting information

This article contains supporting information.

## Conflict of interest

The authors declare that they have no conflicts of interest with the contents of this article.
