# Supplementary material for: Mitochondrial tRNA processing defects reprogram mitochondrial and cellular homeostasis
Source: J Biol Chem. 2025 Jun 3;301(7):110334. doi: 10.1016/j.jbc.2025.110334 (PMC12269583; doi:10.1016/j.jbc.2025.110334)
Supplement: Supplementary Materials [file mmc1.pdf]

SUPPLEMENTARY INFORMATION

Supplemental Figures S1, 2, 3

Supplemental Table 1

A

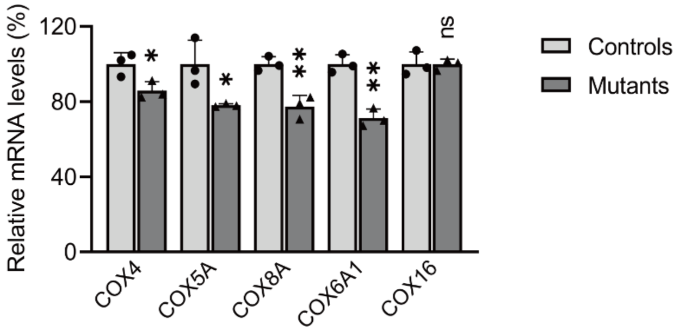

Supplemental Figure S1. Quantification of mRNA levels of COX4, COX5A, COX8A, COX6A1 and COX16 using RT-qPCR, related to Figure 1. Total cellular RNAs were obtained from various cell lines using TRIzol reagent (Invitrogen, 15596026) and reverse transcribed into cDNA using PrimeScript™ RT Master Mix (Takara, RR036A). RT-qPCR was performed on the Applied Biosystems 7900HT Fast Real-Time PCR System. These data were analyzed using the 7900 System SDSRQ Manager and relative gene expressions were calculated using the $2^{-\triangle\triangle Ct}$ method using GAPDH as a housekeeping gene. The calculations were based on three independent determinations. The error bars indicate two standard error of the mean (SEM) of the means. *p* indicates the significance, according to the t-test, of the differences between mutant and control cell lines. *$P < 0.05$; **$P < 0.01$; ***$P < 0.001$; ****$P < 0.0001$; ns, not significant.

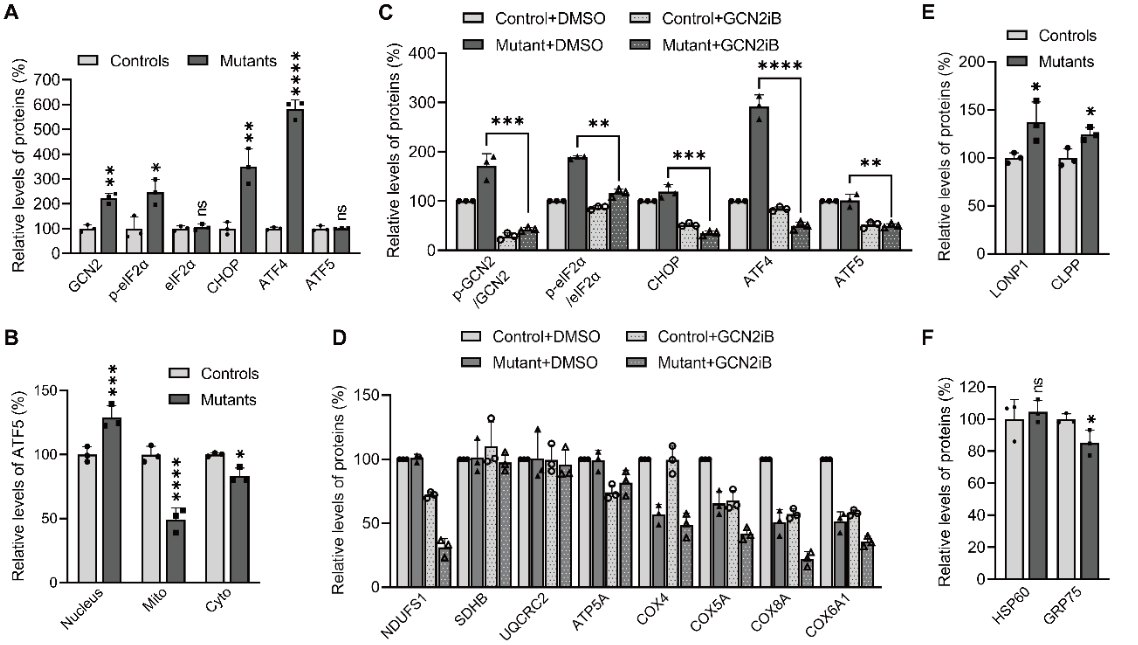

Supplemental Figure S2. Quantification of UPR^{mt}-related proteins, mitochondrial proteases and chaperones, related to Figure 5. (A) Quantification of UPR^{mt}-related proteins GCN2, p-eIF2α, eIF2α, CHOP, ATF4 and ATF5 from three mutant and three control cybrids. (B) Relative levels of ATF5 in the nucleus, mitochondria (Mito) and cytosol (Cyto) fractions between wild type and control cybrids. (C) Proteins involved in GCN signaling (GCN2, p-GCN2, p-eIF2α, eIF2α, CHOP, ATF4 and ATF5) in control (C17.2) and mutant (III-3-2) cybrids after 1 μM GCN2iB or DMSO treatment. (D) Proteins involved in OXPHOS (NDUFS1, SDHB, UQCRC2, ATP5A, COX4, COX5A, COX8A, COX6A1) in control (C17.2) and mutant (III-3-2) cybrids after 1 μM GCN2iB or DMSO treatment. (E, F) Quantification of mitochondrial proteases Lonp1 and Clpp (E) and mitochondrial chaperones HSP60 and GRP75 (F) among six cell lines. Average relative each polypeptide content per cell was normalized to the average content per cell of GAPDH in each cell line. The values for the mutant cell lines are expressed as percentages of the values for the control cell lines. The calculations were based on three independent determinations. The error bars indicate two standard deviations (SD) of the means. *P* indicates the significance, according to the t-test, of the differences between mutant and control cell lines. *$P < 0.05$; **$P < 0.01$; ***$P < 0.001$; ****$P < 0.0001$; ns, not significant.

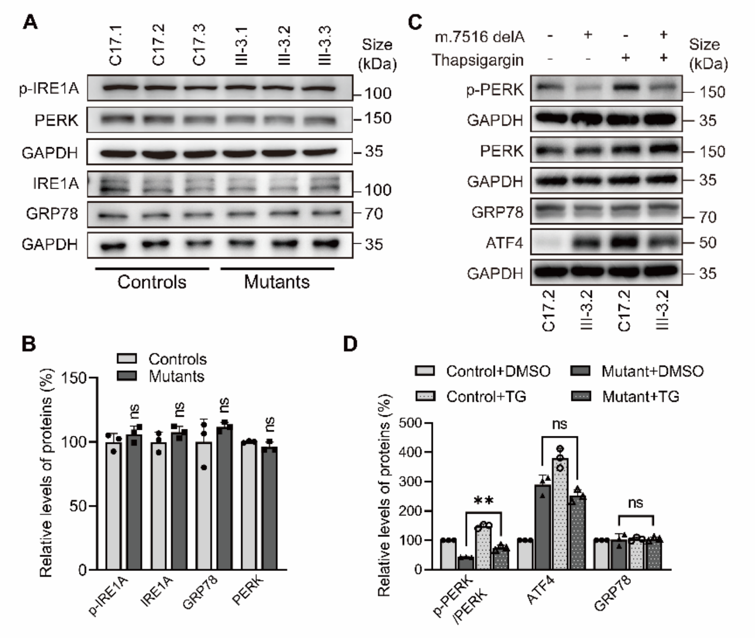

Supplemental Figure S3. Western blot analysis of UPR^{ER} related proteins. Twenty micrograms of total cellular proteins from various cell lines were electrophoresed through a denaturing polyacrylamide gel, electroblotted, and hybridized with antibodies for IRE1A, p-IRE1A, PERK, p-PERK, GRP78 and ATF4, respectively. (A) Western blot analysis of IRE1A, p-IRE1A, PERK and GRP78 in three control and three mutant cybrids. (B) Quantification of IRE1A, p-IRE1A, PERK and GRP78 among these cell lines. (C)Western blot analysis of PERK, p-PERK, ATF4 and GRP78 in one control cyrbids (C17.2) and mutant cybrids (III-3.2) in the presence and absence of thapsigargin. (D Quantification of PERK, p-PERK, ATF4 and GRP78 in one control cyrbids (C17.2) and mutant cybrids (III-3.2) in the presence and absence of thapsigargin (TG). Average relative each polypeptide content per cell was normalized to the average content per cell of GAPDH in each cell line. The values for the mutant cell lines are expressed as percentages of the values for the control cell lines. The calculations were based on three independent determinations. The error bars indicate two standard deviations (SD) of the means. *P* indicates the significance, according to the t-test, of the differences between mutant and control cell lines. *$P < 0.05$; **$P < 0.01$; ***$P < 0.001$; ****$P < 0.0001$; ns, not significant.

Supplemental Table 1

REAGENT or RESOURCE	SOURCE	IDENTIFIER
Antibodies		
Mouse monoclonal anti-LAMP1	Abcam	Cat#ab25630; RRID: AB_470708
Mouse monoclonal anti-SQSTM1/P62	Abcam	Cat#ab56416; RRID: AB_945626
Rabbit monoclonal anti-ATF5	Abcam	Cat#ab184923; RRID: AB_2800462
Rabbit monoclonal anti-GRP78	Abcam	Cat#ab108615; RRID: AB_10890641
Rabbit monoclonal anti-PERK	Abcam	Cat#ab229912; RRID: AB_ 2941805
Rabbit monoclonal anti-SDHC	Abcam	Cat#ab155999; RRID: AB_2810989
Rabbit monoclonal anti-Vinculin	Abcam	Cat#ab129002; RRID: AB_11144129
Mouse monoclonal anti-PINK1	Abcepta	Cat#AW5456; RRID: AB_2924295
Rabbit polyclonal anti-ATG12	Abcepta	Cat#AP1816a; RRID: AB_2059363
Rabbit polyclonal anti-ATG3	Abcepta	Cat#AP1807b; RRID: AB_2059247
Rabbit polyclonal anti-ATG7	Abcepta	Cat#AP1813c; RRID: AB_2062350
Rabbit monoclonal anti-β-Actin	Abclonal	Cat#AC038; RRID: AB_2863784
Rabbit monoclonal anti-Phospho-PERK-T982	Abclonal	Cat#AP1501
Rabbit polyclonal anti-GCN2	Abclonal	Cat#A2307; RRID: AB_2862992
Rabbit polyclonal anti-NDUFA10	Abclonal	Cat#A10123; RRID: AB_2757648
Rabbit polyclonal anti-NDUFS2	Abclonal	Cat#A12858; RRID: AB_2861678
Rabbit polyclonal anti-OPA1	Abclonal	Cat#A9833; RRID: AB_2770723
Rabbit polyclonal anti-PRKN/Parkin	Abclonal	Cat#A0968; RRID: AB_2757487
HRP-labeled goat anti-rabbit IgG	Beyotime	Cat#A0208; RRID: AB_2892644
HRP-labeled goat anti-mouse IgG	Beyotime	Cat#A0216; RRID: AB_2860575
Rabbit polyclonal anti-ATF4	Cell Signaling	Cat#11815; RRID: AB_2616025
Rabbit monoclonal anti-CASP7	Cell Signaling	Cat#12827; RRID: AB_2687912
Rabbit polyclonal anti-BCL-XL	Cell Signaling	Cat#2762; RRID: AB_10694844
Rabbit polyclonal anti-CHOP	Cell Signaling	Cat#2895; RRID: AB_2089254
Rabbit polyclonal anti-EIF2A	Cell Signaling	Cat#9722; RRID: AB_2230924
Rabbit polyclonal anti-HA-Tag	Cell Signaling	Cat#3724; RRID: AB_1549585
Rabbit polyclonal anti-Phospho-DRP1 (Ser616)	Cell Signaling	Cat#4494; RRID: AB_11178659
Rabbit polyclonal anti-Phospho-DRP1 (Ser637)	Cell Signaling	Cat#6319; RRID: AB_10971640
Rabbit polyclonal anti-IRE1 alpha	Novus	Cat# NB100-2324; RRID: AB_10000972
Rabbit polyclonal anti-Phospho-IRE1 alpha (Ser724)	Novus	Cat# NB100-2323; RRID: AB_10145203
Mouse monoclonal anti-CYTC	Proteintech	Cat#66264-1-Ig; RRID: AB_2716798
Mouse monoclonal anti-MFF	Proteintech	Cat#66527-1-Ig; RRID: AB_2881890
Mouse monoclonal anti-GAPDH	Proteintech	Cat#60004-1-Ig; RRID: AB_2107436
Rabbit phosphor-EIF2AK4(Thr899) recombinant antibody	Proteintech	Cat#82835-3-RR
Rabbit polyclonal anti-AFG3L2	Proteintech	Cat#14631-1-AP; RRID: AB_2242420
Rabbit polyclonal anti-ATP5A1	Proteintech	Cat#14676-1-AP; RRID: AB_2061761
Rabbit polyclonal anti-ATP5C1	Proteintech	Cat#10910-1-AP; RRID: AB_2877740
Rabbit polyclonal anti-ATPAF1	Proteintech	Cat#18016-1-AP; RRID: AB_2243352
Rabbit polyclonal anti-ATPB	Proteintech	Cat#17247-1-AP; RRID: AB_2061878
Rabbit polyclonal anti-BAX	Proteintech	Cat#50599-2-Ig; RRID: AB_2061561
Rabbit polyclonal anti-BCL2L13	Proteintech	Cat#16612-1-AP; RRID: AB_1850928
Rabbit polyclonal anti-CASP3/P17/P19	Proteintech	Cat#19677-1-AP; RRID: AB_10733244
Rabbit polyclonal anti-CASP9/P35/P10	Proteintech	Cat#10380-1-AP; RRID: AB_2068632

Rabbit polyclonal anti-CLPP	Proteintech	Cat#15698-1-AP; RRID: AB_2245115
Rabbit polyclonal anti-COX16	Proteintech	Cat#19425-1-AP; RRID: AB_10666854
Rabbit polyclonal anti-COX4	Proteintech	Cat#11242-1-AP; RRID: AB_2085278
Rabbit polyclonal anti-COX5A	Proteintech	Cat#11448-1-AP; RRID: AB_2085429
Rabbit polyclonal anti-COX6A1	Proteintech	Cat#11460-1-AP; RRID: AB_2085445
Rabbit polyclonal anti-COX8A	Proteintech	Cat#15368-1-AP; RRID: AB_10697832
Rabbit polyclonal anti-CYC1	Proteintech	Cat#10242-1-AP; RRID: AB_2090144
Rabbit polyclonal anti-DRP1	Proteintech	Cat#12957-1-AP; RRID: AB_2093525
Rabbit polyclonal anti-EIF2AK2	Proteintech	Cat#18244-1-AP; RRID: AB_2246451
Rabbit polyclonal anti-FIS1	Proteintech	Cat#10956-1-AP; RRID: AB_2102532
Rabbit polyclonal anti-FOXRED1	Proteintech	Cat#24595-1-AP; RRID: AB_2879629
Rabbit polyclonal anti-GRP75	Proteintech	Cat#14887-1-AP; RRID: AB_2120458
Rabbit polyclonal anti-HSP60	Proteintech	Cat#15282-1-AP; RRID: AB_2121440
Rabbit polyclonal anti-Lamin B1	Proteintech	Cat#12987-1-AP; RRID: AB_2136290
Rabbit polyclonal anti-LC3B	Proteintech	Cat#18725-1-AP; RRID: AB_2137745
Rabbit polyclonal anti-MFN1	Proteintech	Cat#13798-1-AP; RRID: AB_2266318
Rabbit polyclonal anti-MFN2	Proteintech	Cat#12186-1-AP; RRID: AB_2266320
Rabbit polyclonal anti-MUL1	Proteintech	Cat#16133-1-AP; RRID: AB_2147111
Rabbit polyclonal anti-NDUFS1	Proteintech	Cat#18443-1-AP; RRID: AB_10699875
Rabbit polyclonal anti-OPTN	Proteintech	Cat#10837-1-AP; RRID: AB_2156665
Rabbit polyclonal anti-Phospho-EIF2S1 (Ser51)	Proteintech	Cat#28740-1-AP; RRID: AB_2881204
Rabbit polyclonal anti-SDHB	Proteintech	Cat#10620-1-AP; RRID: AB_2285522
Rabbit polyclonal anti-TOM20	Proteintech	Cat#11802-1-AP; RRID: AB_2207530
Rabbit polyclonal anti-UQCC2	Proteintech	Cat#25781-1-AP; RRID: AB_2880237
Rabbit polyclonal anti-UQCRC2	Proteintech	Cat#14742-1-AP; RRID: AB_2241442
Rabbit polyclonal anti-UQCRFS1	Proteintech	Cat#18443-1-AP; RRID: AB_10699875
Rabbit polyclonal anti-VDAC1	Proteintech	Cat#55259-1-AP; RRID: AB_10837225
Rabbit polyclonal anti-BNIP3	Sangon Biotech	Cat#D121876
Mouse monoclonal anti-NIX	Santa Cruz	Cat#sc-166332; RRID: AB_2066782
Alexa Fluor 488 rabbit anti-mouse IgG	Yeason	Cat#33906ES60
Alexa Fluor 488 goat anti-rabbit IgG	Yeason	Cat#33106ES60
Alexa Fluor 594 goat anti-mouse IgG	Yeason	Cat#33212ES60
Alexa Fluor 594 goat anti-rabbit IgG	Yeason	Cat#33112ES60
Alexa Fluor 647 goat anti-rabbit IgG	Yeason	Cat#33113ES60
Chemicals, peptides, and recombinant proteins		
Nitrotetrazolium blue chloride	Sangon Biotech	A610379; CAS: 298-83-9
Sodium succinate	Sangon Biotech	A610889; CAS: 150-90-3
Phenazine methosulfate	Sangon Biotech	A610361; CAS: 299-11-6
DAB	Sangon Biotech	A600140; CAS: 868272-85-9
$ATP{\cdot}Na_2$	Sangon Biotech	A600020; CAS: 34369-07-8
Fluoromount	Sigma-Aldich	Cat#F4680
Phenylmethanesulfonyl fluoride	Sigma-Aldich	P7626; CAS: 329-98-6
Digitonin	Sigma-Aldich	D141; CAS: 11024-24-1
NADH	Roche	10107735001; CAS: 606-68-8
Cytochrome c	Sigma-Aldich	C2506; CAS: 9007-43-6
Triton X-100	Sigma-Aldich	T9284; CAS: 9036-19-5
Tween 20	Sigma-Aldich	P7949; CAS: 9005-64-5

MitoTracker Red CMXRos	Thermofisher	Cat#M7512
TEMED	Thermofisher	17919; CAS: 110-18-9
DAPI	Thermofisher	62248; CAS: 28718-90-3
HBSS	Thermofisher	Cat#14175095
Hieff Trans® in vitro siRNA/miRNA Transfection Reagent	Yeason	Cat#40806ES01
Hieff® qPCR SYBR Green Master Mix (No Rox)	Yeason	Cat#11201ES08
Hieff Trans® Liposomal Transfection Reagent	Yeason	Cat#40802ES
Critical commercial assays		
BCA assay	Beyotime	Cat#P0009
Annexin V-FITC Apoptosis Detection Kit	Beyotime	Cat#C1062
Experimental models: Cell lines		
C17.1, C17.2, C17.3, III-3.1, III-3.2 and III-3.3 cybrids	Xiao et al., 2020	N/A
Recombinant DNA		
pCMV_GFP-LC3	This paper	N/A
pCMV_HA-UB	This paper	N/A
pCMV_mCherry-EGFP-LC3	This paper	N/A
Software and algorithms		
Prism 8	GraphPad	https://www.graphpad.com/
ImageJ	NIH	https://imagej.nih.gov/ij/

Supplemental Table 1. Reagents and resources used in this study. This table comprehensively lists antibodies, chemicals, recombinant DNA constructs, cell lines, and software tools employed in the study. For antibodies, catalog numbers (Cat#) and Research Resource Identifiers (RRIDs) are provided. Chemical compounds include CAS registry numbers. Cybrid cell lines (C17.1, III-3.1, etc.) were generated as described in Xiao et al., 2020. Custom plasmids were constructed specifically for this work. Commercial software tools are listed with download links.
